# Clinical study of renal impairment in patients with propylthiouracil-induced small-vessel vasculitis and patients with primary ANCA-associated small-vessel vasculitis

**DOI:** 10.3892/etm.2013.1065

**Published:** 2013-04-15

**Authors:** XIAOZHI CAO, WEIYUAN LIN

**Affiliations:** 1 Department of Cardiology, Fuzhou General Hospital, Nanjing Command, PLA, Fuzhou 350025;; 2 Renal Department of Internal Medicine, The Second Affiliated Hospital of Fujian Medical University, Quanzhou 362000, P.R. China

**Keywords:** propylthiouracil, anti-neutrophil cytoplasmic antibody, vasculitis, renal disease

## Abstract

The aim of this study was to compare renal impairment in patients with propylthiouracil (PTU)-induced small-vessel vasculitis and patients with primary anti-neutrophil cytoplasmic antibody (ANCA)-induced small-vessel vasculitis. The study compared the pathology and clinical conditions of 10 patients with PTU-associated small-vessel vasculitis and 18 patients with primary ANCA-associated small-vessel vasculitis. All patients showed manifestations of renal impairment. Compared with the primary ANCA-induced small-vessel vasculitis, patients with PTU-induced small-vessel vasculitis were mostly female (P<0.05) and deleloped the disease at a younger age (P<0.05). They had a higher positive rate of perinuclear anti-neutrophil cytoplasmic antibody (p-ANCA; P<0.05), lower 24 h proteinuria content, lower serum creatinine (P<0.05) and milder histopathological lesions (P<0.05). A number of them had gross hematuria (P<0.05). They rarely used hormone and cytotoxic drugs (P<0.05) during treatment and had a better prognosis (P<0.05). In conclusion, PTU-induced small-vessel vasculitis has a milder pathology and clinical manifestations with a better prognosis.

## Introduction

Primary vasculitis is a systemic disease of unknown etiology (1–3). The main pathological features are vascular wall inflammation and fibrinoid necrosis. It is closely related with the anti-neutrophil cytoplasmic antibody (ANCA) (4) and is known as the primary ANCA-associated small-vessel vasculitis (primary AAV). According to the Chapel Hill Consensus Conference in 1994, AASV (antibody-associated systemic vasculitis) includes granulomatosis with polyangiitis (GPA, previously known as Wegener’s granulomatosis), microscopic polyangiitis (MPA) and eosinophilic granulomatosis with polyangiitis (EGPA, formerly known as Churg-Strauss vasculitis or Churg-Strauss) (5–7). AASV involves the systemic small vessels and multiple organs. Research shows that the renal impairment is more frequently observed and more severe in AASV, and approximately half of the patients have this manifestation in the early stage of the disease. Propylthiouracil (PTU) is a drug frequently used to treat hyperthyroidism (8) and its side effects are frequently reported. In recent years, PTU-induced ANCA-associated small-vessel vasculitis, also known as PTU-associated small-vessel vasculitis, is increasingly reported (9,10). Renal impairment is the most frequent and severe symptom observed. The majority of previous reports indicate that PTU-associated small-vessel vasculitis has a better prognosis than the ANCA-induced variant but pathological and clinical comparisons of the two are uncommon. This study analyzes the similarities and differences in the renal impairment of both forms from the pathological and clinical aspects. The results should assist in diagnosis and treatment.

## Subjects and methods

### Research subjects

Patients with PTU-associated small-vessel vasculitis and patients with primary AAV diagnosed from 2002 to 2011 were selected as the research subjects. The diagnostic criteria for PTU-associated small-vessel vasculitis were as follows: i) The patient had manifestations of vasculitis, which coincided with taking PTU; ii) Positive serum ANCA; iii) Other diseases that cause vasculitis were excluded, such as malignant tumor and infection. The diagnostic criteria for primary AAV referred to related diagnostic criteria provided by the Chapel Hill Consensus Conference in 1994 and the American College of Rheumatology (ACR). All patients had manifestations of renal impairment, such as hematuria, proteinuria, renal insufficiency and hypertension and their renal biopsy was suggestive of vasculitis. This study was conducted in accordance with the declaration of Helsinki and with the approval of the Ethics Committee of the Second Affiliated Hospital of Fujian Medical University. Written informed consent was obtained from all participants.

### Clinical and laboratory examination

According to the analysis of the general characteristics of the patients (age and gender) and renal impairment (including gross hematuria, 24 h proteinuria content and serum creatinine), perinuclear anti-neutrophil cytoplasmic antibody (p-ANCA) and cytoplasmic anti-neutrophil cytoplasmic antibody (c-ANCA) were detected using indirect immunofluorescence (IIF) and myeloperoxidase (MPO)-ANCA and proteinase 3 (PR3)-ANCA were detected using enzyme-linked immunosorbent assay (ELISA).

### Renal biopsy

All patients underwent percutaneous renal biopsy to observe through routine light microscopy, immunofluorescence and electron microscopy the number of glomerular crescents (including cellular crescents, fibrocellular crescents and fibrous crescents), glomerular capillary loop necrosis and renal tubulointerstitial lesions.

### Treatment

PTU-induced small-vessel vasculitis was treated based on the following principles (11): i) immediate withdrawal of PTU; ii) if necessary, separate use of hormones or use of hormones in combination with cyclophosphamide depending on the state of illness. Primary AAV was treated referring to previous reports in the literature.

The curative effect was classified into complete, partial and no remission as per criteria in the literature (12).

### Statistical analysis

SPSS version 14.0 was used for statistical analysis with the statistical data expressed as mean ± SD. P<0.05 was considered to indicate a statistically significant result.

## Results

### General characteristics

This study included 10 patients with PTU-induced small-vessel vasculitis and 18 patients with primary AAV (including 16 patients with MPA, 1 patient with GPA and 1 patient with EGPA). Compared with primary AAV, the patients with PTU-induced small-vessel vasculitis were mostly female and young when falling ill (P<0.05, Table I).

### Laboratory examination

Compared with primary AAV, the patients with PTU-induced small-vessel vasculitis had higher positive rate of p-ANCA (P<0.05, Table II), as was indicated by the indirect immunofluorescence. ELISA showed that 1 patient with PTU-induced small-vessel vasculitis demonstrated positive MPO-ANCA and PR3-ANCA but none of the patients with primary AAV presented double positive, showing that the patients with PTU-induced small-vessel vasculitis had a higher proportion of dysimmunity. A few patients with such disease had gross hematuria (P<0.05, Table II) and all of the patients with such disease had lower 24 h proteinuria content, lower serum creatinine (P<0.05, Table II) and milder renal impairment.

### Renal biopsy

Compared with primary AAV, the patients with PTU-induced small vasculitis had a lower proportion of crescents (P<0.05, Table III), a lower proportion of glomerular capillary loop necrosis (P<0.05), a lower proportion of interstitial fibrosis (P<0.05, Table III), a lower proportion of arteriolar fibrinoid necrosis (P<0.05) and a lower proportion of inflammatory cell infiltration (P<0.05, Table III.). These data suggested that the patients with PTU-induced small-vessel vasculitis had milder histopathological lesions.

### Treatment and outcomes

Among the patients with PTU-induced small-vessel vasculitis, 3 were not administered any hormone or cytotoxic drug following the withdrawal of PTU, 6 of them were administered hormone only and 1 of them was administered hormone in combination with cyclophosphamide. All patients with primary AAV were administered hormone and cytotoxic drugs, 2 were treated with blood purification (13) and the difference was statistically significant (Z=−4.7, P=0.00). All patients were followed up for 9–24 months (median follow-up time of 15 months), which showed that among patients with PTU-induced small-vessel vasculitis, 7 presented complete remission, 3 presented partial remission and none had no remission; in patients with primary AAV, 5 patients presented complete remission, 9 patients presented partial remission and 5 patients had no remission. The comparison showed that the difference between the two groups was statistically significant (Z=−2.3, P=0.02).

## Discussion

In 1992, Stankus and Johnson reported the first case of PTU-induced small-vessel vasculitis (14). Since then several cases of PTU-induced small-vessel vasculitis have been reported (15). PTU induces the generation of ANCA. According to this report, 20–64% of patients administered PTU progressed to positive ANCA and ∼4–6.5% of patients may progress to vasculitis (16). It has different pathogeneses and different clinical and pathological characteristics from primary AAV. This paper systematically compared the clinical and pathological characteristics of renal impairment of both conditions. The results show that patients with PTU-induced small-vessel vasculitis are mostly young and female with a higher positive rate of p-ANCA, milder clinical and pathological characteristics, easier treatment and better prognosis.

Both the present study and a previous study (16) show that patients with PTU-induced small-vessel vasculitis are mostly young and female, and patients with primary AAV are mostly old-aged without gender difference. The cause may be that the patients with hyperthyroidism are mainly young and female and are mainly treated using oral drugs, ^131^I or surgery. ^131^I and surgery may involve drawbacks such as large trauma and more complications. As a result, these patients are mainly treated with oral drugs.

ANCA is the first autoantibody proven to be associated with vasculitis. The target antigens of ANCA include various ingredients in the neutrophil cytoplasm, mainly PR3 and MPO (17,18). In the two groups of patients, p-ANCA is more frequently observed than c-ANCA. Primary AAV is not a disease but a group of diseases. c-ANCA and its target antigen PR3-ANCA are more frequently observed in patients with GPA, while p-ANCA and its target antigen MPO-ANCA are more frequently observed in patients with MPA and EGPA (3). In the present study, the total number of patients with MPA and patients with EGPA was 41 while the number of GPA patients was 7. This may explain why p-ANCA and its target antigen MPO-ANCA are more frequently observed in patients with primary AAV. p-ANCA is more frequently observed in patients with PTU-induced small-vessel vasculitis and p-ANCA and its target antigen MPO-ANCA may be induced by PTU (19). The specific mechanism needs to be elucidated. In this study, positive PR3-ANCA and MPO-ANCA were only seen in patients with PTU-induced small-vessel vasculitis and were not seen in patients with primary AAV. The mechanism may be associated with the polyclonal activation of B-lymphocytes. In addition to the target antigens PR3-ANCA and MPO-ANCA, other ANCA antigens may also be detected in patients taking PTU. This phenomenon was not found in patients with primary AAV and the degree of renal impairment was not related to the amount of ANCA target antigen (19–21). The specific mechanism needs further investigation.

In the present study, compared with primary AAV, patients with PTU-induced small-vessel vasculitis have milder renal impairment and better prognosis. This may be associated with prompt removal of contributing factors of the disease, as well as the low age of patients in this group. Some studies show that old age is a predictor for prognosis of primary AAV (22). In conclusion, patients with PTU-induced small-vessel vasculitis have milder renal impairment than those with primary AAV, and this may also be associated with better prognosis.

## Figures and Tables

**Table I:** Comparison of the general characteristics of both groups.

	PTU-associated small-vessel vasculitis (n=10)	Primary AAV (n=18)	Statistical data	P-value
Number of female patients	9 (90.0%)	7 (38.9%)	-	0.01
Age of onset	39.2±8.4	60.4±10.1	t=−5.6	0.00

PTU, propylthiouracil; AAV, ANCA-associated small-vessel vasculitis; ANCA, anti-neutrophil cytoplasmic antibody.

**Table II:** Comparison of the laboratory examination of both groups.

	PTU-associated small-vessel vasculitis (n=10)	Primary AAV (n=18)	Statistical data	P-value
Indirect immunofluorescence of ANCA				
Number of patients with p-ANCA	10 (100.0%)	10 (55.6%)	-	0.02
Number of patients with c-ANCA	0 (00.0%)	8 (44.4%)	-	0.02
ELISA of ANCA				
Number of patients with MPO-ANCA only	8 (80.0%)	11 (61.1%)		
Number of patients with PR3-ANCA only	1 (10.0%)	7 (38.9%)	χ^2^=0.63	0.42
Number of patients with double positive	1 (10.0%)	1 (10.0%)	0 (0.0%)	
Number of patients with gross hematuria	1 (10.0%)	10 (55.6%)	-	0.04
24 h proteinuria content (mg/24 h)	578.5±119.7	1541.7±334.7	t=−11.0	0.00
Creatinine (*μ*mol/l)	159.9±50.9	567.6±112.1	t=−13.1	0.00

PTU, propylthiouracil; AAV, ANCA-associated small-vessel vasculitis; ANCA, anti-neutrophil cytoplasmic antibody; p-ANCA, perinuclear anti-neutrophil cytoplasmic antibody; c-ANCA, cytoplasmic anti-neutrophil cytoplasmic antibody; ELISA, enzyme-linked immunosorbent assay; MPO, myeloperoxidase; PR3, proteinase 3.

**Table III:** Comparison of the renal biopsy of both groups.

	PTU associated small-vessel vasculitis (n=10)	Primary AAV (n=18)	Statistical data	P-value
Proportion of cellular crescents (%)	11.9±5.8	45.3±9.9	t=−9.7	0.00
Proportion of fibrocellular crescents (%)	2.5±1.8	20.1±7.0	t=−7.8	0.00
Proportion of fibrous crescents (%)	3.9±2.2	16.3±3.9	t=−9.3	0.00
Glomerular capillary loop necrosis	2 (20.0%)	12 (66.7%)	−	0.04
Interstitial fibrosis	1 (10.0%)	10 (55.6%)	−	0.04
Arteriolar fibrinoid necrosis	1 (10.0%)	11 (61.1%)	−	0.04
Inflammatory cell infiltration	1 (20.0%)	13 (72.2%)	−	0.00

PTU, propylthiouracil; AAV, ANCA-associated small-vessel vasculitis; ANCA, anti-neutrophil cytoplasmic antibody.
